# Human Leukocyte Antigen and Red Blood Cells Impact Umbilical Cord Blood CD34^+^ Cell Viability after Thawing

**DOI:** 10.3390/ijms20194875

**Published:** 2019-09-30

**Authors:** Diana Vanegas, Cristian-Camilo Galindo, Iván-Aurelio Páez-Gutiérrez, Lorena-Xiomara González-Acero, Pavel-Tiberio Medina-Valderrama, Juan-Camilo Lozano, Bernardo Camacho-Rodríguez, Ana-María Perdomo-Arciniegas

**Affiliations:** 1 Specialized researcher, Cord Blood Bank, Instituto Distrital de Ciencia, Biotecnología e Innovación en Salud. Cra. 32 # 12-81, Bogotá 111611, Colombia; dmvanegas@idcbis.org.co (D.V.); ccgalindoun@gmail.com (C.-C.G.); ipaez@idcbis.org.co (I.-A.P.-G.); lxgonzalez@idcbis.org.co (L.-X.G.-A.); ptmedina@idcbis.org.co (P.-T.M.-V.); jclozanoc@unal.edu.co (J.-C.L.); 2 Director, Instituto Distrital de Ciencia, Biotecnología e Innovación en Salud. Cra. 32 # 12-81, Bogotá 111611, Colombia; bacamacho@idcbis.org.co; 3 Scientific leader, Cord Blood Bank, Instituto Distrital de Ciencia, Biotecnología e Innovación en Salud. Cra. 32 # 12-81, Bogotá 111611, Colombia

**Keywords:** umbilical cord blood, hematopoietic stem cell transplantation, human leukocyte antigen, cell viability, thawing

## Abstract

Hematopoietic progenitor cell (HPC) transplantation is a treatment option for malignant and nonmalignant diseases. Umbilical cord blood (UCB) is an important HPC source, mainly for pediatric patients. It has been demonstrated that human leukocyte antigen (HLA) matching and cell dose are the most important features impacting clinical outcomes. However, UCB matching is performed using low resolution HLA typing and it has been demonstrated that the unnoticed mismatches negatively impact the transplant. Since we found differences in CD34^+^ viability after thawing of UCB units matched for two different patients (*p* = 0.05), we presumed a possible association between CD34^+^ cell viability and HLA. We performed a multivariate linear model (*n* = 67), comprising pre-cryopreservation variables and high resolution HLA genotypes separately. We found that pre-cryopreservation red blood cells (RBC), granulocytes, and viable CD34^+^ cell count significantly impacted CD34^+^ viability after thawing, along with HLA-B or -C (*R*^2^ = 0.95, *p* = 0.01; *R*^2^ = 0.56, *p* = 0.007, respectively). Although HLA-B*40:02 may have a negative impact on CD34^+^ cell viability, RBC depletion significantly improves it.

## 1. Introduction

Autologous or allogeneic hematopoietic progenitor cell (HPC) transplantation has been clinically used for immune reconstitution in patients with hematological malignant and nonmalignant diseases [1]. Along with bone marrow and mobilized peripheral blood, umbilical cord blood (UCB) is an important HPC source with reduced viral infection transmission risk and less graft-versus-host disease (GVHD) incidence [2,3]. However, UCB-transplanted patients exhibit delayed granulocyte and platelet engraftment compared to the adult sources and therefore an increased risk of non-relapse mortality [2,3,4]. Total nucleated cell (TNC) dose and human leukocyte antigen (HLA) matching between donor and recipient are crucial to obtain better clinical outcomes [5,6,7]. 

The importance of TNC might be associated to its correlation with total CD34^+^ cells [8,9,10,11], which has been proposed as a transplantation outcome predictor by some authors [4,11,12]. However, volume reduction and cryopreservation processes decrease TNC and CD34^+^ cell number and viability after thawing [12,13], although it has been demonstrated that CD34^+^ cells are highly resistant to cryopreservation with dimethyl sulfoxide (DMSO) [14,15,16]. 

Regarding HLA, a lower restriction in donor and patient matching is firmly established for UCB transplantation compared to adult cell sources due to less incidences of chronic GVHD and presumed lesser T-cell immune maturity [1,2,3]. Matching for UCB transplantation has been traditionally performed at antigenic level for HLA-A and -B and at allelic level for HLA-DRB1 [17]. However, several authors have raised important questions regarding typing resolution in UCB donors and its impact on transplant outcomes [17,18,19,20,21,22,23,24]. The UCB donor selection algorithm has an implied disadvantage compared to adult unrelated [23] or haploidentical donors and this may impact on transplantation clinical outcomes with this source.

The public umbilical cord blood bank in Colombia assessed confirmatory testing results for at least three UCB units per patient during the first year performing clinical searches. In several cases, all matching UCB units to a particular patient were in a narrow range of CD34^+^ cell viability percentage. Since the only connection between these selected UCB units was HLA matching with the patient, we evaluated if there was an association between HLA genotypes and CD34^+^ cell viability after thawing. We found that HLA-B and HLA-C genotypes, along with UCB pre-cryopreservation variables (red blood cells, viable CD34^+^ cell count, and granulocytes percentage), impact on CD34^+^ cell viability after thawing, according to multivariate linear models. Moreover, red blood cell (RBC) depletion before cryopreservation significantly improved CD34^+^ cell viability after thawing. 

## 2. Results

### 2.1. CD34^+^ Cell Viability after Thawing was Assessed Using an Automated Gating Method

**Figure 1:**
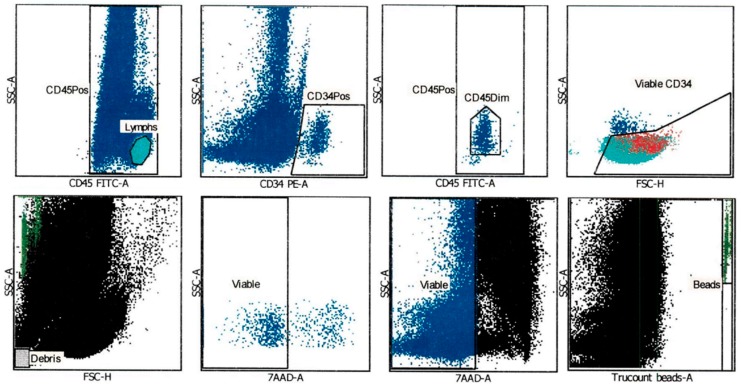
BD FACSCanto clinical software v2.4 standard gating strategy to determine stem cell count and viability with the BD stem cell enumerator kit. The image shows a representative sample of a confirmatory testing assessment by flow cytometry. From left to right in the upper panels, automated gating strategy was performed as follows: lymphocyte population selection (first panel, left), followed by CD34^+^ cell population (second panel). In the CD34^+^ cell population, CD45^dim^ population was selected, which represents the most primitive CD34^+^ cells (third panel). Location of the CD34^+^ cells (in red) inside the lymphocyte population (fourth panel) indicated viability. For viability assessment the gating strategy was performed as follows: from left to right in the bottom panels: debris were excluded from the viability and cell count analysis (first panel). Previously selected CD34^+^CD45^dim^ and total CD45^+^ viability was assessed through negative 7-AAD signal in the second and third panels. In the fourth panel, beads were excluded (by APC^+^ signal) to allow cell count extrapolation.

UCB banking was established in 1995 [13], including processing and cryopreservation procedures. In 1996, the International Society of Hematotherapy and Graft Engineering established a Stem Cell Enumeration Committee, which validated and published flow cytometry guidelines to determine CD34^+^ cell count using a reliable and reproducible assay in peripheral blood samples [25]. Later, the protocol was adapted for UCB samples, including a viability dye (7-amino actinomycin D; 7-AAD), an ammonium chloride buffer for red blood cell lysis, and an ISHAGE-adapted gating strategy [26], which was used in all viability and cell count assessments in this work. A representative sample of the gating strategy is shown in Figure 1. This staining protocol and gating strategy were used in both pre-cryopreservation and after thawing UCB units. First, lymphocytes population was selected by CD45 high expression, while primitive cells population was selected by CD34 expression. From the latter one, CD45^dim^ population was counted as the most primitive. This population was also shown to be located inside the previously selected lymphocytes. Debris were excluded from the analysis. Viable CD45^+^ and CD34^+^ cells were determined through negative 7-AAD signal. A known number of count beads were separated in a gate, to extrapolate CD45^+^ and CD34^+^ cell count. (Figure 1). 

### 2.2. Patient-Dependent CD34^+^ Cell Viability after Thawing

Since there is no national donor registry in Colombia, the public UCB bank is currently performing a donor search for pediatric transplantation candidate patients in the UCB units’ inventory. UCB units bear three attached segments (distal, medial, and proximal according to their proximity to the unit), which are considered representative samples to verify cell viability, cell recovery, and clonogenic potency (CLONE) after thawing. This process is denominated confirmatory testing and the protocol was previously standardized in the bank for clinical purposes [27]. Confirmation of the distal segment as a representative sample of the UCB unit was also performed to guarantee reliability of the results [28]. 

**Figure 2:**
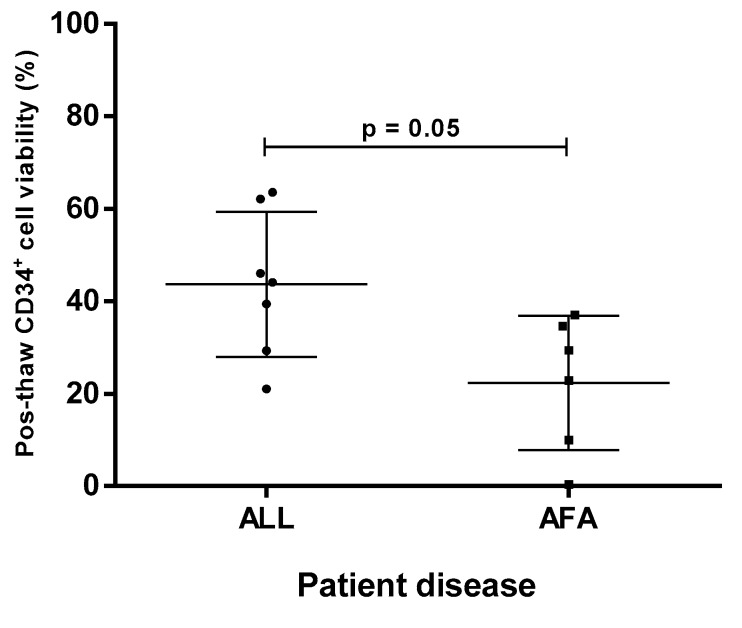
Comparison of CD34^+^ cell viability percentage from confirmatory testing after distal segment thawing. Seven umbilical cord blood (UCB) units matched with the acute lymphoblastic leukemia (ALL) patient and six UCB units matched with the aplastic/Fanconi anemia patient (AFA) patient. All units exhibited a 4/6 or 5/6 HLA match considering the three-locus algorithm (HLA-A, HLA-B, HLA-DRB1).

Once a clinical search for a patient is received, the search software (MatchIDCBIS) lists all possible donors (UCB units) according to HLA matching (HLA-A and -B at allelic level and –DRB1 at molecular level) and sorts them by the highest cell dose. During the first year of clinical searches, we performed confirmatory testing for the best three resulting UCB units per patient and found a particular case in which all CD34^+^ cell viability data were below our acceptability criteria (aplastic/Fanconi anemia patient; AFA). Confirmatory testing of the remaining UCB units for this patient were similar. We therefore selected another patient (acute lymphoblastic leukemia; ALL) with a similar amount of confirmatory testing results and compared CD34^+^ cell viability after thawing (Figure 2).

Figure 2 shows that CD34^+^ cell viability after thawing was significantly lower in the confirmatory testing for the AFA patient (mean = 26.1%) than the ALL patient (mean = 44.1%). The only common feature between all tested UCB units per patient was HLA typing, which in all cases exhibited a 4/6 or 5/6 matching, always determined by HLA-A and HLA-B at allelic level and HLA-DRB1 at molecular level. Therefore, it is possible that HLA is somehow affecting CD34^+^ cell viability after thawing.

### 2.3. HLA Genotype per Locus Does Not Impact CD34^+^ Cell Viability after Thawing

To determine if HLA was directly impacting CD34^+^ cell viability after thawing in confirmatory testing results, we performed a simple linear regression model with HLA genotypes per locus (molecular level) as the explanatory variable and CD34^+^ cell viability after thawing as the dependent variable. A total of 67 confirmatory testing data from UCB unit attached segments were available and used for the linear model. Descriptive statistics of the sample are shown in Table 1. 

It was not possible to include the five loci genotypes simultaneously in the linear model, therefore we tested genotypes per locus obtaining five independent linear models. However, HLA genotype data were not enough to explain the CD34^+^ cell viability after thawing, in either HLA-A (*p* = 0.68), HLA-B (*p* = 0.51), HLA-C (*p* = 0.22), HLA-DRB1 (*p* = 0.32), or HLA-DQB1 (*p* = 0.06).

### 2.4. Pre-Cryopreservation RBC and HLA-B and -C Impact on CD34^+^ Cell Viability after Thawing

**Figure 3:**
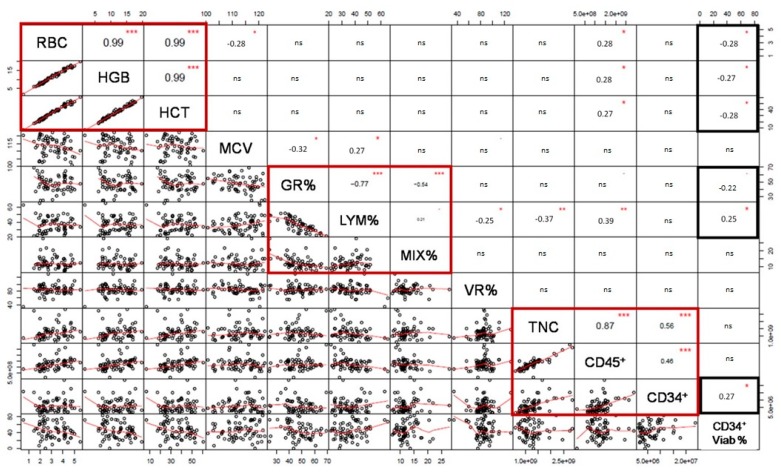
Correlation matrix using pre-cryopreservation variables and CD34+ cell viability after thawing (CD34+ Viab %). The matrix shows significance levels in the opposite squares to the correlated factors, r value was shown, ns means no significant correlation. Red squares exhibit significantly correlated variables. Black squares show variables correlated with CD34+ cell viability post-thawing. RBC= red blood cells (10^6^ /µL); HGB = hemoglobin (g/dl); HCT = hematocrit (%); MCV = media corpuscular volume (fl); GR% = percentage of granulocytes (%); %LYM = percentage of lymphocytes (%); MIX% = percentage of mixed cells (%); VR% = volume reduction efficiency (%); TNC = total nucleated cells; CD45^+^ = viable CD45^+^ cell count; CD34^+^ = viable CD34^+^ cell count and CD34^+^ Viab% = viability percentage of CD34^+^ cells after thawing (%). RBC, HGB, HCT, MCV, GR%, LYM%, MIX%, and TNC were determined by an automated hematology analyzer in the UCB units before cryopreservation. Volume reduction percentage (VR%) was calculated comparing initial TNC (before volume reduction) with pre-cryopreservation TNC. Viable CD45^+^ and CD34+ cell count were determined by flow cytometry.

Although HLA genotypes did not exclusively explain CD34^+^ cell viability after thawing by a linear model, it was possible that other variables during UCB processing contributed to the observed differences when comparing patient-associated searches [29]. A multivariate linear model is an alternative to determine which variables are impacting on CD34^+^ cell viability after thawing. The selection of the continuous independent variables for the multivariate model was assessed through a correlation matrix including pre-cryopreservation cellular variables. The matrix allowed the identification of variables that did not covariate to include in the model. Evaluated pre-cryopreservation variables were: RBC density, hemoglobin (HGB), hematocrit (HCT), mean corpuscular volume (MCV), granulocytes percentage (GR%), lymphocytes percentage (LYM%), mixed cells percentage (MIX%), volume reduction percentage (VR%), TNC, viable CD45^+^, and CD34^+^ cell count. Since CD34^+^ cell viability after thawing is also a continuous variable and the dependent variable in the linear model, it was included in the matrix to verify any possible relation with the pre-cryopreservation variables (Figure 3).

As expected, all RBC-associated variables (RBC, HGB, HCT) exhibited a direct significant correlation (red box; *R*^2^ = 0.99, *p* < 0.001), while GR% was inversely correlated with LYM% and MIX% (red box; *R*^2^ = −0.77, *p* < 0.001 and *R*^2^ = −0.54, *p* < 0.001). LYM% and MIX% were also directly correlated, although in a lesser extent (*R*^2^ = 0.21, *p* = 0.05). Finally, TNC, viable CD45^+^, and CD34^+^ cell count were also directly correlated with statistical significance (TNC vs. viable CD45^+^ cell count *R*^2^ = 0.87, *p* < 0.001; TNC vs. viable CD34^+^ cell count *R*^2^ = 0.56, *p* > 0.001; and viable CD45^+^ cell count vs. viable CD34^+^ cell count *R*^2^ = 0.46, *p* > 0.001).

With regard to CD34^+^ cell viability after thawing (CD34^+^ Viab% in Figure 3), there was an inversely significant correlation with all RBC-associated variables (black box, HGB, and HCT) (*R*^2^ = 0.26, *p* > 0.01) and also with GR% (*R*^2^ = −0.23, *p* = 0.05). Pre-cryopreservation viable CD34^+^ cell count was also significantly correlated to CD34^+^ cell viability after thawing (*R*^2^ = 0.27, *p* < 0.01). A variable of each covariation group in the matrix (pre-cryopreservation RBC, GR%, and viable CD34^+^ cell count or TNC) along with HLA genotypes for each locus were selected as independent variables for the multivariate linear model to explain CD34^+^ cell viability after thawing. To verify that the range of dependent and independent variables was wide enough to determine a comparison, we assessed the frequency distribution of each one (Figure S1). CD34^+^ cell viability after thawing was widely distributed from 0% to 83.2% (Table 1 and Figure S1), pre-cryopreservation RBC ranged from 0.5 to 5.5 × 10^6^ cells/µL, pre-cryopreservation GR% from 25% to 70%, pre-cryopreservation TNC from 6 to 28 × 10^8^ cells, and pre-cryopreservation viable CD34^+^ cell count from 2 to 24 × 10^6^ cells.

A general multivariate linear model was performed using the selected variables with each HLA genotype per locus. Since pre-cryopreservation TNC is a more commonly considered characteristic than pre-cryopreservation viable CD34^+^ cell count by physicians, two analyses were performed separately with each variable, along with pre-cryopreservation RBC, GR%, and HLA. As shown in Table 2, CD34^+^ cell viability after thawing was significantly explained (95%) by a linear model with HLA-B genotype, pre-cryopreservation RBC, GR%, and viable CD34^+^ cell count (*p* = 0.014) and 56% was explained with the same independent variables and HLA-C genotype (*p* = 0.007). The HLA-C model is well adjusted, either with pre-cryopreservation viable CD34^+^ cell count or TNC, while the HLA-B model is only significant with pre-cryopreservation viable CD34^+^ cell count. Data including HLA-A, -DRB1, and –DQB1 genotypes did not significantly adjust a linear model (Table 2).

Complete multivariate linear model results with HLA-B and HLA-C genotypes exhibiting estimates and individual variable statistical significance are shown in Tables S1–S3. According to the individual variable statistical significance, a higher pre-cryopreservation RBC dose negatively affected CD34^+^ cell viability after thawing in both models (*p* = 0.015 in the HLA-B model and *p* = 0.001 in the HLA-C model). In the HLA-C model, GR% negatively affected CD34^+^ cell viability after thawing (*p* = 0.012), while pre-cryopreservation viable CD34^+^ cell count and TNC did not. In the HLA-B model, pre-cryopreservation viable CD34^+^ cell count significantly affected CD34^+^ cell viability after thawing (*p* = 0.030), while pre-cryopreservation GR% did not. Multicollinearity in the linear models were determined by a variance inflation factors test. There was no covariation between the variables in the HLA-C model, however, in the HLA-B model, pre-cryopreservation RBC, GR% and viable CD34^+^ cell count exhibited multicollinearity.

In the HLA-B model, HLA-B*40:02g allele was present as part of several significant genotypes negatively impacting CD34^+^ cell viability after thawing. However, this could be due to the high frequency of this allele in our population, as it has been previously reported (15.9% of the individuals have the allele) [30]. HLA-B*35:43 and HLA-B*40:02 were the most common alleles in the typified samples from our 1,416 UCB donors (allele frequencies of 8.65% and 8.44%, respectively) [30]. In the HLA-C model, all individually significant genotypes negatively affected CD34^+^ cell viability after thawing. Post-hoc analyses were not performed.

### 2.5. Allele-Related Differences in CD34^+^ Cell Viability after Thawing

**Figure 4:**
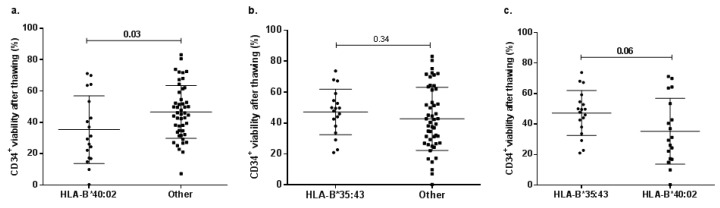
CD34^+^ cell viability percentage after thawing in UCB units bearing particular HLA-B alleles that were part of the statistically significant genotypes in the multivariate linear model. (**a**) There were statistically significant differences in CD34^+^ cell viability between UCB units with HLA-B*40:02 (*n* = 18) and without the allele (*n* = 49). (**b**) There were no significant differences in CD34^+^ cell viability between UCB donors with HLA-B*35:43 (*n* = 18) and donors without it (*n* = 49). (**c**) There were no significant differences in CD34^+^ cell viability between UCB units with HLA-B*40:02 and with HLA-B*35:43. None of the UCB donors bared both alleles in a genotype.

In order to assess if particular HLA alleles might be associated with an after thawing CD34^+^ cell viability decrease; a comparison between UCB units (donors) with and without alleles was performed. Allele selection depended on their presence as part of the significant genotypes in the multivariate linear models and frequency in the UCB donor population [30]. Evaluated alleles were HLA-B*35:43, HLA-B*40:02, HLA-C*03:04, and HLA-C*03:05 (Figure 4).

CD34^+^ cell viability after thawing of UCB units bearing HLA-B*40:02 was significantly lower than those without it (HLA-B*40:02 mean = 35.3%, other alleles mean = 46.6%, *p* = 0.03; Figure 4a), while in the HLA-B*35:43 case, there were no significant differences (HLA-B*35:43 mean = 47.3%, other alleles mean = 42.8%, *p* = 0.34; Figure 4b). Considering the high frequency of both alleles, we compared CD34^+^ cell viability after thawing from UCB donors with HLA-B*35:43 and HLA-B*40:02 (HLA-B*35:43 mean = 47.3%, HLA-B*40:02 = 35.3%, *p* = 0.06; Figure 4c). However, there were no statistically significant differences. There were no statistically significant differences in HLA-C alleles either (*p* > 0.05).

### 2.6. RBC Depletion Increases CD34^+^ Cell Viability after Thawing

**Figure 5:**
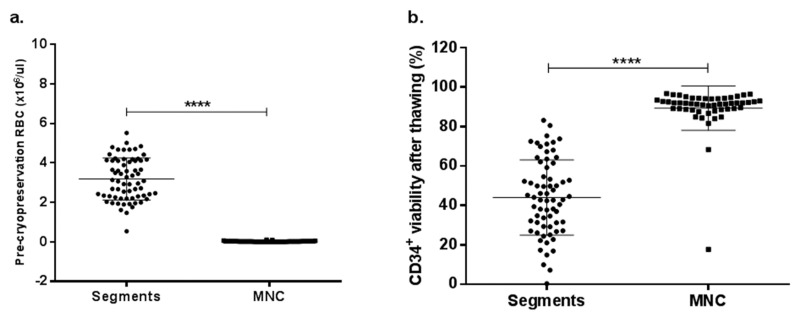
RBC content and CD34^+^ cell viability after thawing comparison between UCB unit segments and RBC-depleted mononuclear cells (MNC). (**a**) Pre-cryopreservation RBC difference between two volume reduction methods: UCB traditional processing and density-gradient MNC isolation. (**b**) CD34^+^ cell viability after thawing differences between two volume reduction methods: UCB traditional processing and density-gradient MNC isolation.

Since RBC content was the most impactful continuous variable in the multivariate linear models with HLA-B and –C, a mononuclear cells (MNC) isolation method to completely deplete RBC before cryopreservation was performed to compare CD34^+^ cell viability after thawing with the clinical UCB units. A total of 52 UCB units were collected and processed by a density gradient method as described in the methods section. Figure 5 shows pre-cryopreservation RBC (×10^6^ cells/µL) data from the UCB unit distal segments used for the multivariate linear model (*n* = 67) compared to the MNC isolated by a density gradient (*n* = 52). There were statistically significant differences in RBC content between UCB unit segments (range 0.5–5.5 ×10^6^ cells/µL; mean = 3.2 ×10^6^ cells/µL) and RBC-depleted MNC (range 0.01–0.1 ×10^6^ cells/µL; mean = 0.03 ×10^6^ cells/µL; *p* < 0.0001; Figure 5a).

All samples were thawed using the previously mentioned standardized protocol [27]. CD34^+^ cell viability after thawing exhibited a statistically significant difference between UCB unit distal segment and RBC-depleted MNC (*p* < 0.0001). While CD34^+^ cell viability from segments with a higher pre-cryopreservation RBC dose ranged from 0.3% to 83.2% (mean = 44%), RBC-depleted MNC ranged from 17.7% to 96.8% (mean = 89.4%), showing a critical improvement.

## 3. Discussion

It has been demonstrated that a mismatch two or more alleles out of eight (considering four loci) between UCB donor and patient negatively impacts the overall survival after transplantation, while one or more mismatches are associated with a higher probability of graft failure [18,23,24,31]. According to multivariate linear model analysis results, particular HLA genotypes may have a negative effect on CD34^+^ cell viability after thawing, but only when pre-cryopreservation cellular variables were included (RBC, viable CD34^+^ cell count, and GR%). Nevertheless, HLA genotypes as exclusive independent variables, although typed in high resolution, did not fit the linear model.

We tested five different HLA loci (HLA-A, -B, -C, -DRB1, and –DQB1), from which only HLA-B and –C exhibited a significant fit to the linear model along with the mentioned pre-cryopreservation variables. Interestingly, when performing the diagnostic proofs, we found multicollinearity between the continuous variables in the HLA-B model, but not in the HLA-C, nor when the linear model was performed without any HLA. We presume that this particular class I HLA locus may set up a particular relation between these variables.

As for the pre-cryopreservation variables included in the multivariate linear model, RBC content exhibits a higher negative impact on CD34^+^ cell viability after thawing compared to GR%, viable CD34^+^ cell count or TNC. We evaluated the individual impact of RBC content using an alternative UCB volume reduction method to completely deplete these cells. CD34^+^ cell viability after thawing was almost doubled when RBC were removed and results were consistent for almost all UCB units. RBC reduction was established in UCB banking in order to optimize liquid nitrogen storage and consequently, decrease costs. Broxmeyer et al. [32] stated that CD34^+^ cell loss by volume reduction was undesirable for transplant success, but traditional practices include RBC and plasma depletion under the rationale of collecting a large blood volume (therefore TNC) to overcome this issue [12,13,33]. Although it has not been assessed in UCB, Assmus et al. [34] demonstrated that a 24 h co-culture of bone marrow MNC with RBC from the same donor impaired in vitro cell viability, invasion capacity, SDF-1 chemotactic response and clonogenic capacity, and in vivo functional response using a mouse model.

In UCB, the depletion of RBC content before cryopreservation increased CD34^+^ cell viability after thawing, however, there were also low viabilities. Thus, RBC content had a significant impact on CD34^+^ cell viability, probably along with the presence of particular HLA alleles. It is important to note that HLA-B*40:02 was the most frequent allele present in statistically significant genotypes in the multivariate model, mainly with a negative effect. The HLA-B*40:02 allele is highly present in our population (8.44%) [30] and has a significant effect on CD34^+^ cell viability compared to other alleles.

Previous studies have demonstrated an association between HLA-B*40:02 and acquired aplastic anemia [35,36,37,38], where the auto-antigen presentation caused a T-cell-mediated cytotoxicity, and in consequence, allele-expressing CD34^+^ cells were eliminated [35,38,39]. This does not necessarily mean that merely the presence of a particular allele determines whether a UCB unit will survive after thawing or not, but might be an important feature to consider, mainly with aplastic anemia patients who are candidates for transplantation. Regarding the HLA-C in our donor population, the most frequent alleles found in haplotype with HLA-B*40:02 were HLA-C*03:05 and -C*03:04 [30]. We found that all HLA-C significant genotypes negatively impacted CD34^+^ cell viability in the linear model, whereas some of the HLA-B genotypes exhibited positive estimates. However, due to the two locus linkage, it is possible that the negative effect of HLA-B is related to particular genes of HLA-C as well.

Multivariate analysis results suggest that the CD34^+^ cell viability after thawing is affected by the presence of RBC, GR%, and certain HLA-B/-C alleles. The effect of all these variables can be due to an unfavorable environment inherent to the pre-cryopreservation or thawing processes [40]. It is possible that granulocyte cell content release, along with RBC, affects CD34^+^ cell viability after thawing, with a potentiated effect on particular HLA genotypes, but further experimental approaches are needed. If these factors are as important as hypothesized, HLA-B and -C, along with pre-cryopreservation RBC, GR%, and viable CD34^+^ cell count might be considered for UCB selection. As Eapen et al. have suggested [18], a new donor selection algorithm including not only additional HLA allele matching but also UCB unit cellular features may improve clinical outcomes in UCB transplantation patients.

## 4. Materials and Methods

### 4.1. Umbilical Cord Blood Collection and Volume Reduction

All UCB units were collected from healthy donors at public hospitals in Bogotá, Colombia after signing an informed consent form reviewed by the Ethical Committee of the District Secretary of Health. As previously described [41], UCB units were obtained from single-birth, full-term deliveries. The exclusion criteria were maternal infections or viral diseases, absence of prenatal care and low neonatal weight. We performed an interview to determine maternal and newborn medical and family history. All UCB units collected were transported to the bank to determine TNC in an automated hematocytometer (Sysmex, XN1000, Roche, Basilea, Switzerland). From 2014 to 2016, only UCB units with over 10 × 10^8^ TNC were selected for volume reduction and cryopreservation. Since 2016, the threshold was increased to 12.5 × 10^8^. Volume reduction was performed in a closed system (Biosafe, Lake Geneva, Switzerland) with hydroxyethyl starch (HES 6%, Grifols S.A., Barcelona, Spain) for RBC sedimentation.

The alternative open volume reduction method for complete RBC depletion was performed using a density gradient medium (Lymphoprep™, Stemcell technologies, Vancouver, BC, Canada). The collected UCB volume was initially centrifugated at 800× *g* for 20 min to collect plasma and isolate buffy coat. Buffy coat was washed using phosphate-buffered saline 1X (PBS 10X, Thermo Fisher Scientific, Waltham, MA, USA) seeded over Lymphoprep^TM^ medium for MNC isolation, centrifuging at 500× *g* for 30 min. Meanwhile, plasma was heat-inactivated at 56 °C for 40 min. Finally, MNC were washed three times with PBS 1X centrifuging at 700× *g* for 10 min and cell density was determined by an automated hematology analyzer.

Quality control tests included flow cytometry to determine CD45^+^ and CD34^+^ cell count and viability percentages. Samples were stained following the instructions in the diagnostic tools stem cell enumerator kit (Cat 344563) and Trucount tubes (Cat 340334) (BD, Franklin Lakes, NJ, USA). All samples were processed in a FACSCanto II cytometer (BD, Franklin Lakes, NJ, USA), which is the appropriate instrument for these clinical purposes according to the kit manual. Before assessing cell count and viability from the samples, the instrument was calibrated using BD FACS™ 7-color setup beads (Cat 335775). Setup and analysis was performed using the BD FACSCanto clinical software v2.4 following BD Stem Cell Enumeration Application Guide for BD FACSCanto II Flow Cytometers (BD, Franklin Lakes, NJ, USA). The software automatically uses a gating algorithm to identify stem cells (CD34^+^) and does not allow major modifications. We daily measured CD34^+^ cell count controls provided every month by Becton Dickinson and verified that the obtained results were between the established ranges. Once all controls were accurate, UCB samples from donors were acquired.

All samples were stained in a Trucount (BD, Franklin Lakes, NJ, USA) tube using 100 µL of UCB with 20 µL of the BD Stem Cell reagent (anti-CD45-FITC/anti-CD34-PE in phosphate-buffered saline, bovine serum albumin, and sodium azide) and 20 µL of 7-AAD to identify dead cells incubated 20 min at room temperature. RBC were depleted using an ammonium chloride lysis buffer 1X for 10 min at room temperature which was also included in the kit. Samples were acquired immediately after finishing incubation. Software acquisition and analysis methods are based on the Clinical and Laboratory Standards Institute H42-A2 approved guideline. All UCB units included in the inventory exhibited higher TNC than 7 × 10^8^ and over 1.5 × 10^6^ CD34^+^ viable cells after volume reduction. All UCB units positive for microbiological contamination, infectious diseases, or hemoglobinopathies were excluded from the clinical use inventory.

### 4.2. Cord Blood Cryopreservation and Attached Distal Segment Thawing

After volume reduction, 6 mL of DMSO/Dextran40 (CryoSure, WAK-Chemie Medical GmbH, Steinbach, Germany) were added to the UCB unit by an automated closed system using a controlled time rate (0.5 mL/min). UCB units were maintained at 4 °C during DMSO addition. Cryopreservation was performed with a freezing curve ranging from 4 to 0 °C (4 °C/min), to –10 °C (1 °C/min), to –50 °C (20 °C/min), to –18 °C (15 °C/min), to –40 °C (1 °C/min), to –60 °C (2 °C/min), and to –80 °C (3 °C/min) in a CryoMed Freezer (Thermo Scientific, Waltham, Massachusetts, USA). After reaching –80 °C, UCB units were stored in the vapor phase of liquid nitrogen (–190 °C). The temperature of UCB units was monitored constantly in the storage tank.

In the alternative open volume reduction method for complete RBC depletion, MNC cell suspension was cryopreserved with DMSO/Dextran40 in 1.5 mL vials with 60 × 10^6^ cells per vial. Vial number depended on the total MNC available per unit. Cryopreservation was performed overnight in a –80 °C freezer with final storage in the vapor phase of liquid nitrogen (–190 °C).

Donor search was carried out by the UCB bank search coordinator using the MatchIDCBIS software through an established protocol. Segment confirmatory testing was performed on the better-matching UCB donor units per search, as indicated by the results. Use of genetic data from search results (UCB units) was authorized by the Research Ethics Committee of Fundación Hospital Pediátrico La Misericordia (HOMI) in Bogotá, Colombia on May 31 of 2018 (Acta No. 014 CEI 41-18). All analyzed data were obtained directly from the UCB processing procedure, before cryopreservation and after thawing.

For confirmatory testing, attached distal segments were extracted in a cryocart (CHART MVE Medical, Ball Ground, GA, USA), keeping the temperature below –150 °C during the whole procedure. The segments were thawed in sterile conditions with a previously standardized protocol [28]. Briefly, 100 µL of UCB obtained from the segment were diluted 1/8 in a HES-based media and incubated for 30 min. Flow cytometry was performed as described previously, as well as TNC count. For clonogenic culture assays, 150 CD34^+^ cells/mL of media (Methocult, Stem Cell Technologies, Vancouver, BC, Canada) were seeded per well, with two wells per UCB unit, and cultured for 14 days at 37 °C and 5% of CO_2_. CLONE was calculated as the proportion of colonies formed compared to the 150 CD34^+^ cells seeded. A total of 67 distal segments were thawed for this analysis. Appendix A describes the whole procedure.

### 4.3. Cord Blood HLA Typing

All UCB units in the clinical use inventory (1416) were typed in allele-level resolution for five loci (HLA-A, -B, -C, -DRB1, and -DQB1) in a certified laboratory (Histogenetics, Ossining, NY, USA). Five spots of 25 µL whole blood samples were seeded before volume reduction in protein saver cards (Whatman, Sigma-Aldrich, St. Louis, MI, USA) and sent to Histogenetics for HLA typing by next-generation sequencing (NGS) as described by the laboratory [42]. For further analysis, the allele results were reduced to a four-digit codification with Hapl-o-Mat software [43] and Microsoft Excel, respectively. Graphs of the impact of continuous variables grouped by HLA alleles were obtained in R i386 3.2.2 software [44].

### 4.4. Statistical Analysis

Initial, post-volume reduction, and post-thawing data of 67 distal segments were included in the analysis. Descriptive statistics was determined in GraphPad Prism version 7.00 for Windows (GraphPad Software, La Jolla, CA, USA, www.graphpad.com). The correlation matrix, univariate, and multivariate analyses were performed in R i386 3.2.2 software [44]. Haplotype and genotype frequencies were determined by Hapl-o-Mat [43] and Microsoft Excel, respectively. Graphs of the impact of continuous variables grouped by HLA alleles were obtained in R i386 3.2.2 software [44]. All comparisons between two groups (alleles, RBC content, and RBC impact on CD34+ cell viability after thawing) were obtained by Mann–Whitney U test in GraphPad Prism version 7.00 for Windows (GraphPad Software, La Jolla, CA, USA, www.graphpad.com).

## Figures and Tables

**Table 1:** Descriptive statistics of 67 distal segments used for confirmatory testing. Total nucleated cell (TNC) were determined using an automated hematology analyzer; CD45^+^ and CD34^+^ viable cell count and viability percentage were determined by flow cytometry; and CLONE was determined by cell culture and manual colony count. All UCB units were available for clinical purposes.

Cellular Variables after Thawing							
Variable	*n*	Mean	Median	SD	SEM	Minimum	Maximum
TNC (×10^8^)	65	10.57	9.92	4.51	0.56	3.10	23.30
TNC Recovery (%)	65	85.10	88.10	26.33	3.27	23.70	141.50
Viable CD45^+^ (×10^8^)	67	3.97	3.69	1.65	0.20	0.12	7.99
CD45^+^ Recovery (%)	67	33.82	34.50	14.34	1.75	8.90	71.90
CD45^+^ Viability (%)	60	49.98	49.35	13.38	1.73	5.10	81.0
Viable CD34^+^ (×10^6^)	67	2.64	1.67	2.43	0.30	0.02	13.20
CD34^+^ Recovery (%)	67	40.57	40.20	20.37	2.49	0.60	83.30
CD34^+^ Viability (%)	67	44.02	42.90	19.06	2.33	0.31	83.20
CLONE (%)	64	36.40	36.35	18.10	2.26	0.0	82.30

**Table 2:** General multivariate linear regression model with CD34^+^ viability after thawing as the dependent variable and HLA genotypes, pre-cryopreservation RBC, GR%, and viable CD34^+^ cell count as explanatory variables. There was only statistical significance in the model with HLA-B and HLA-C genotypes. All continuous variables exhibited multicollinearity in the HLA-B model according to the variance inflation factors test.

Multivariate Model (Dependent Variable: CD34^+^ Cell Viability)				
HLA	Explanatory Variables	*R* ^2^	*p*	Multicollinearity
A	RBC, GR%, CD34^+^	0.05	0.449	No
	RBC, GR%, TNC	0.05	0.444	No
B	RBC, GR%, CD34^+^	0.95	0.014	RBC, GR%, CD34^+^
	RBC, GR%, TNC	0.79	0.108	RBC, GR%
C	RBC, GR%, CD34^+^	0.56	0.007	No
	RBC, GR%, TNC	0.55	0.007	No
DRB1	RBC, GR%, CD34^+^	0.26	0.316	%GR, CD34^+^
	RBC, GR%, TNC	0.28	0.298	CNT
DQB1	RBC, GR%, CD34^+^	0.27	0.060	No
	RBC, GR%, TNC	0.26	0.066	No

**Figure A1:**
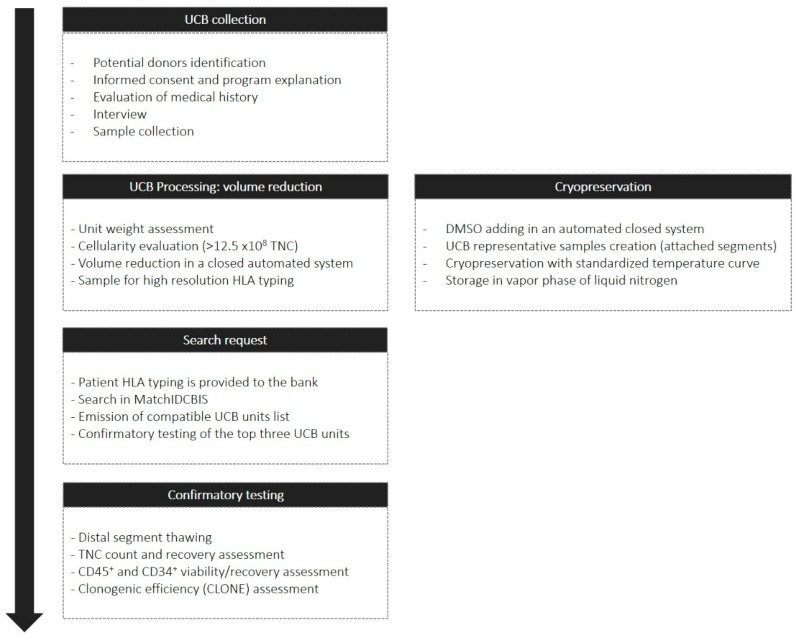
Flow work of UCB collection, volume reduction, cryopreservation, search request, and confirmatory testing in the umbilical cord blood bank.

## Supplementary Materials

Supplementary materials can be found at https://www.mdpi.com/1422-0067/20/19/4875/s1.

## Abbreviations

HPC	Hematopoietic progenitor cell
UCB	Umbilical cord blood
HLA	Human leukocyte antigen
GVHD	Graft vs. host disease
TNC	Total nucleated cell
DMSO	Dimethyl sulfoxide
RBC	Red blood cells
CLONE	Clonogenic efficiency
HGB	Hemoglobin
HCT	Hematocrit
MCV	Mean corpuscular volume
GR%	Granulocytes percentage
LYM%	Lymphocytes percentage
MIX%	Mixed cells percentage
VR%	Volume reduction percentage
