# Supplementary material for: Human Leukocyte Antigen and Red Blood Cells Impact Umbilical Cord Blood CD34+ Cell Viability after Thawing
Source: Int J Mol Sci. 2019 Sep 30;20(19):4875. doi: 10.3390/ijms20194875 (PMC6801469; doi:10.3390/ijms20194875)
Supplement: Supplementary file 1 [file ijms-20-04875-s001.zip › ijms-594019-supplementary/SUPPLEMENTARY MATERIAL FIGS1 AND LEGENDS.docx]

**SUPPLEMENTAL MATERIAL**

**FIGURE S1.**

**
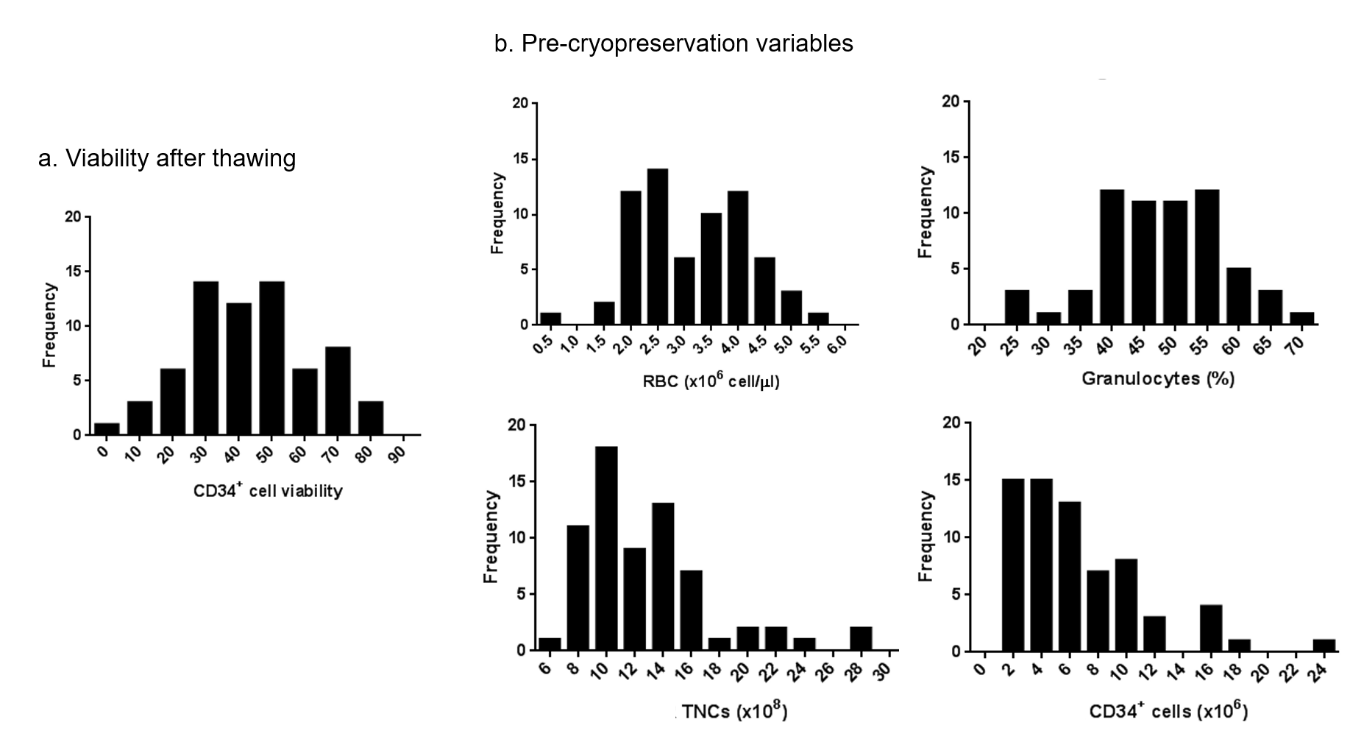
FIGURE LEGENDS TO SUPPLEMENTAL MATERIAL**

**Figure S1.** Frequency distribution of variables considered for the multivariate linear model **a)** CD34^+^ cell viability percentage after thawing which ranges from 0 to 80% **b)** RBC ranges from 0.5 to 6.0 x10^6^ cell/µl, GR% ranges from 20 to 70%, TNC ranges from 6 to 28x10^7^ cells and CD34^+^ranges from 0 to 24x10^6^ cells. We determined all involved variables frequency distribution in order to determine if there is enough range to find any relation in the multivariate model.

**Table S1.** Detailed multivariate linear regression model of CD34^+^ cell viability after thawing showing individual variable estimates and statistical significance. Independent variables included were: HLA-B genotypes in high resolution typing and pre-cryopreservation cellular variables (RBC, GR% and CD34^+^ cell count). Variables were sorted by statistical significance. The allele HLA-B*40:02 is present in five significant genotypes. From the pre-cryopreservation cellular variables, only RBC and CD34^+^ cell count were individually significant in the model.

**Table S2.** Detailed multivariate linear regression model of CD34^+^ cell viability after thawing showing individual variable estimates and statistical significance. Independent variables included were: HLA-C genotypes in high resolution typing and pre-cryopreservation cellular variables (RBC, GR% and CD34^+^ cell count). Variables were sorted by statistical significance. From the pre-cryopreservation variables in this model, only RBC and GR% were individually significant.

**Table S3.** Detailed multivariate linear regression model of CD34^+^ cell viability after thawing showing individual variable estimates and statistical significance. Independent variables included were: HLA-C genotypes in high resolution typing and pre-cryopreservation cellular variables (RBC, GR% and TNC). Variables were sorted by statistical significance. TNC were not statistically significant in the model.
